# Anti-influenza virus activity of extracts from the stems of *Jatropha multifida* Linn. collected in Myanmar

**DOI:** 10.1186/s12906-017-1612-8

**Published:** 2017-02-07

**Authors:** Masaki Shoji, So-Yeun Woo, Aki Masuda, Nwet Nwet Win, Hla Ngwe, Etsuhisa Takahashi, Hiroshi Kido, Hiroyuki Morita, Takuya Ito, Takashi Kuzuhara

**Affiliations:** 1 Laboratory of Biochemistry, Faculty of Pharmaceutical Sciences, Tokushima Bunri University, 180 Yamashiro-cho, Tokushima, 770-8514 Japan; 2 Institute of Natural Medicine, University of Toyama, 2630, Sugitani, Toyama 930-0194 Japan; 3 Department of Chemistry, University of Yangon, Yangon, 11041 Myanmar; 4 Division of Pathology and Metabolome Research for Infectious Disease and Host Defense, Institute for Enzyme Research, University of Tokushima, 3-18-15, Kuramoto-cho, Tokushima 770-8503 Japan

**Keywords:** Anti-influenza, Anti-virus, *Jatropha multifida*, Stem, Herbal medicine

## Abstract

**Background:**

To contribute to the development of novel anti-influenza drugs, we investigated the anti-influenza activity of crude extracts from 118 medicinal plants collected in Myanmar. We discovered that extract from the stems of *Jatropha multifida* Linn. showed anti-influenza activity. *J. multifida* has been used in traditional medicine for the treatment of various diseases, and the stem has been reported to possess antimicrobial, antimalarial, and antitumor activities. However, the anti-influenza activity of this extract has not yet been investigated.

**Methods:**

We prepared water (H_2_O), ethyl acetate (EtOAc), *n*-hexane (Hex), and chloroform (CHCl_3_) extracts from the stems of *J. multifida* collected in Myanmar, and examined the survival of Madin-Darby canine kidney (MDCK) cells infected with the influenza A (H1N1) virus, and the inhibitory effects of these crude extracts on influenza A viral infection and growth in MDCK cells.

**Results:**

The H_2_O extracts from the stems of *J. multifida* promoted the survival of MDCK cells infected with the influenza A H1N1 virus. The EtOAc and CHCl_3_ extracts resulted in similar, but weaker, effects. The H_2_O, EtOAc, and CHCl_3_ extracts from the stems of *J. multifida* inhibited influenza A virus H1N1 infection; the H_2_O extract possessed the strongest inhibitory effect on influenza infection in MDCK cells. The EtOAc, Hex, and CHCl_3_ extracts all inhibited the growth of influenza A H1N1 virus, and the CHCl_3_ extract demonstrated the strongest activity in MDCK cells.

**Conclusion:**

The H_2_O or CHCl_3_ extracts from the stems of *J. multifida* collected in Myanmar demonstrated the strongest inhibition of influenza A H1N1 viral infection or growth in MDCK cells, respectively. These results indicated that the stems of *J. multifida* could be regarded as an anti-influenza herbal medicine as well as a potential crude drug source for the development of anti-influenza compounds.

## Background

In 1918, the Spanish influenza A (H1N1) virus pandemic caused 50 million deaths worldwide [1, 2]. In 2009, influenza A virus originating in swine (H1N1) caused a pandemic, and the avian H5N1 and H7N9 influenza A viruses in China are highly pathogenic to humans [1–4]. Currently, the application of three antiviral medicines known as neuraminidase (NA) inhibitors, oral oseltamivir, zanamivir, and peramivir, is recommended for the treatment of influenza. However, oseltamivir resistance has been detected in some of the 2009-derived H1N1 viruses and the seasonal H1N1 viruses between 2007 and 2009, but little in H3N2 viruses [5]. In the future, zanamivir- and peramivir-resistant strains, similar to oseltamivir-resistant strain, will emerge. Therefore, the development of novel anti-influenza drugs to prevent and control future influenza epidemics and pandemics is desired.

Traditional medicinal plants have been recognized as a rich source of candidate compounds for the development of pharmaceuticals [6, 7]. A large number of natural products and extracts from medicinal plants have been reported to possess anti-influenza virus activity [8–10]. Therefore, many studies have focused on traditional medicinal plants as an important source of candidate compounds for the discovery of novel anti-influenza drugs.

The abundance of medicinal plants in Myanmar has enabled the population to use traditional medicines to maintain their own health and treat various diseases. Thus, to discover sources for novel anti-influenza drugs, we screened extracts from 118 medicinal plants collected in Myanmar to analyze the cell viability of influenza A H1N1 virus (A/PR/8/34)-infected MDCK cells using naphthol blue black staining. We identified six medicinal plants that promoted the survival of influenza A virus-infected cells selected by the criteria described at the Methods section. Of these six plants, the activity of extract from the stems of *Jatropha multifida* Linn (*J. multifida*) was strongly pronounced. *J. multifida*, a member of the family Euphorbiaceae, is a tree of 2–3 m in height, and widely distributed in sub-tropical and tropical areas throughout Asia and Africa [11]. Popularly known as “Say-ma-khan”, it is commonly used as a folk medicine in Myanmar and has been used as a purgative, and against fever, indigestion, colic, wounds, and skin infection [11]. The seed oil, latex, and leaves are effective purgatives and abortifacients, have been used as wound dressings, and for the treatment of neurodermatitis, eczema, and itches [11]. The roots and stems have antimicrobial, antimalarial, antitumor, antileishmanial, and antiulcer activities [11, 12]. In addition, previous phytochemical studies on *J. multifida* reported the presence of cyclic peptides, diterpenoids, and phenolic compounds [11]. However, pharmacological and phytochemical investigations of *J. multifida* stems originating from Myanmar have not yet been conducted, which attracted us to investigate whether extracts from the stems of *J. multifida*, obtained using various solvents, possessed anti-influenza virus activity.

## Methods

### Plant material

The stems of *J. multifida* were purchased from Sandhi Brothers Trading Co. Ltd (Yangon, Myanmar) in November 2015. A voucher specimen (TMPW 28729) was deposited at the Museum of Materia Medica, Analytical Research Center for Ethnomedicines, Institute of Natural Medicine, University of Toyama, Japan.

### Plant extraction

We performed the plant extraction as described previously [13, 14]. In brief, dried stems of *J. multifida* were chopped into small pieces (3.0 kg), which were macerated four times with 70% aqueous EtOH (7 L) in an ultrasonic bath for 90 min at 25 °C. After filtration of the suspension, the resulting solution was evaporated under reduced pressure to yield a crude extract (180 g). The crude extract was suspended in water and partitioned into *n*-hexane (Hex), chloroform (CHCl_3_), and ethyl acetate (EtOAc) fractions, to yield Hex-soluble (12.2 g), CHCl_3_-soluble (11.0 g), and EtOAc-soluble portions (11.0 g), respectively. Finally, the residual aqueous layer was evaporated under reduced pressure to yield a water (H_2_O)-soluble portion (140.8 g). The extracts were stored at a concentration of 10 mg/mL in dimethyl sulfoxide (DMSO).

### Cells

Madin-Darby canine kidney (MDCK) cells were cultured in high-glucose Dulbecco’s modified Eagle’s medium (DMEM; Wako, Osaka, Japan), supplemented with 10% fetal bovine serum (FBS; Life Technologies, CA, USA), 50 units/mL penicillin and 50 μg/mL streptomycin (P/S; Life Technologies), and 4 mM l-glutamine, at 37 °C in the presence of 5% CO_2_ [15].

### Viral strain

This study used the Puerto Rico 8/34 (A/PR/8/34; H1N1) strain of the influenza A virus. Viral titers were determined by immunostaining influenza A viral nucleoprotein (NP) as previously described [16].

### Analysis of cell viability of influenza A virus-infected MDCK cells using naphthol blue black staining

MDCK cells were seeded in a 96-well plate (1 × 10^4^ cells/well). H_2_O, EtOAc, Hex, or CHCl_3_ extracts from the stems of *J. multifida* (0.8-25 μg/mL in DMSO) were mixed with influenza A virus in 10% FBS-supplemented growth medium at a multiplicity of infection (MOI) of 10, and then incubated for 30 min at 37 °C in the presence of 5% CO_2_. DMSO (0.008-0.5%) and (+)-(*S*)-bakuchiol (0.8-25 μM in DMSO) were used as negative and positive controls, respectively, for the inhibition of influenza A viral infection [15]. The mixture was added to the cells, which were then incubated for 4 days at 37 °C in the presence of 5% CO_2_. After incubation, the cells were stained using naphthol blue black as previously described [15, 17]. The viable cells in each well were stained blue, while dead cells remained unstained. The selection criteria for the six plants are more than 50% cell survival at 96 h after the viral infection with the concentration of 50 μg/mL.

### Thiazolyl blue tetrazolium bromide (MTT) assay

MDCK cells were seeded in each well of a 96-well plate (1 × 10^4^ cells/well). H_2_O, EtOAc, Hex, or CHCl_3_ extracts (12.5-100 μg/mL) were prepared in DMSO (12.5 μg/mL, 0.125%; 25 μg/mL, 0.25%; 50 μg/mL, 0.5%; 100 μg/mL, 1%) and mixed with infection medium (DMEM supplemented with 1% bovine serum albumin [BSA; Wako, Osaka, Japan], P/S, and 4 mM l-glutamine). The mixture was added to the cells, which were then incubated for 24, 72, or 96 h at 37 °C in the presence of 5% CO_2_. After incubation, cell viability was determined using the MTT cell proliferation assay as previously described [15].

### Immunofluorescence staining of influenza A virus-infected cells

MDCK cells were seeded in a 96-well plate (1 × 10^4^ cells/well). H_2_O, EtOAc, Hex, or CHCl_3_ extracts from the stems of *J. multifida* (3.1-25 μg/mL) or (+)-(*S*)-bakuchiol (3.1-25 μM) were mixed with influenza A virus at a MOI of 0.1 in the infection medium and incubated for 30 min at 37 °C in the presence of 5% CO_2_. DMSO (0.031-0.25%) was used as the negative control. Each mixture was added to the cells and incubated for 24 h at 37 °C in the presence of 5% CO_2_. The cells were fixed with 4% paraformaldehyde in PBS for 30 min at 4 °C and then permeabilized by the addition of 0.3% Triton X-100 for 20 min at 25 °C. A mouse antibody for the detection of the NP of A/PR/8/34 (FluA-NP 4 F1; SouthernBiotech, AL, USA) was used as the primary antibody. Alexa Fluor488-conjugated goat anti-mouse IgG (H + L) antibody (Life Technologies, CA, USA) was used as the secondary antibody. Cell nuclei were then stained using diamidino-2-phenylindole (DAPI; Life Technologies). The wells were photographed using a fluorescence microscope (BIOREVO BZ-9000, Keyence, Osaka, Japan), and the percentage of influenza A NP-positive cells per DAPI-positive cells were calculated based on measurements recorded with BZ-H1C software (Keyence).

### Influenza A viral growth assay

To explore whether the extracts from the stems of *J. multifida* affected viral growth in pre-infected cells, MDCK cells were seeded in a 24-well plate (1 × 10^5^ cells/well). The cells were infected with A/PR/8/34 (MOI; 0.001) in infection medium for 1 h at 37 °C in the presence of 5% CO_2_. The infected cells were washed prior to the addition of H_2_O, EtOAc, Hex, or CHCl_3_ *J. multifida* extracts (12.5 or 25 μg/mL in 0.5% DMSO) to the cells in infection medium supplemented with 3 μg/mL l-tosylamido-2-phenyl ethyl chloromethyl ketone (TPCK)-treated trypsin (Sigma-Aldrich). DMSO (0.5%) and ribavirin (50 μM in 0.5% DMSO) were the negative and positive controls, respectively, for the inhibition on influenza A viral growth [18]. The cells were then incubated for 24, 48, or 72 h at 37 °C in the presence of 5% CO_2_. Cell culture media were collected from each well at predetermined time points. Viral titers (plaque forming units per mL [PFU/mL]) were determined as previously described [15].

### Statistical analysis

All results were expressed as the mean ± the standard error of the mean (SEM). Differences between more than two groups were analyzed for statistical significance by using one-way analysis of variance (ANOVA). Values of *p* < 0.05 were considered statistically significant.

## Results

### Extracts from the stems of *J. multifida* increased the survival of influenza A viral-infected MDCK cells

**Fig. 1:**
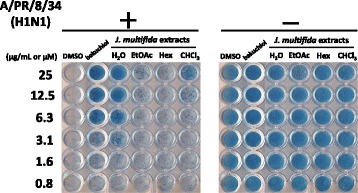
Effect of extracts from the stems of *J. multifida* on the viability of MDCK cells infected with influenza A H1N1 virus. H_2_O, EtOAc, Hex, and CHCl_3_ extracts from the stems of *J. multifida* (0.8-25 μg/mL in DMSO) were mixed with or without (virus-) influenza A H1N1 virus (A/PR/8/34) (MOI; 10), and added to MDCK cells. DMSO (0.008-0.5%) and (+)-(*S*)-bakuchiol (bakuchiol; 0.8-25 μM in DMSO) were used as negative and positive controls, respectively, for the inhibition of influenza A viral infection. After incubation for 4 days, cell viability was determined by naphthol blue black staining. Data are representative of three independent experiments, and the results were found to be reproducible

To evaluate the anti-influenza viral activity of extracts from the stems of *J. multifida*, we first examined the survival of influenza A virus-infected MDCK cells after treatment with the H_2_O, EtOAc, Hex, or CHCl_3_ extracts from the stems of *J. multifida.* As shown in Fig. 1, cells exposed to DMSO and infected with A/PR/8/34 were not stained. However, cells treated with 3.1-25 μM (+)-(*S*)-bakuchiol or 3.1-25 μg/mL H_2_O extract and infected with A/PR/8/34 were stained blue. Cells exposed to 25 μg/mL EtOAc or 12.5-25 μg/mL CHCl_3_ extract and infected with A/PR/8/34 were also weakly stained blue (Fig. 1).

**Fig. 2:**
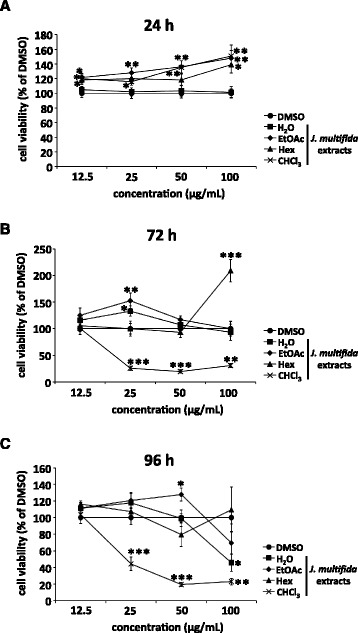
Toxicity of extracts from the stems of *J. multifida* to MDCK cells. H_2_O, EtOAc, Hex, and CHCl_3_ extracts from the stems of *J. multifida* (12.5-100 μg/mL) in DMSO (concentrations of DMSO: 12.5 μg/mL, 0.125%; 25 μg/mL, 0.25%; 50 μg/mL, 0.5%; 100 μg/mL, 1%) were added to the MDCK cells. Cell viabilities were determined via MTT assay after 24 h (*n* = 6 each) (**a**), 72 h (*n* = 6 each) (**b**), and 96 h (*n* = 6 each) (**c**). Data are the mean ± SEM representative of two independent experiments. *p < 0.05; **p < 0.01; ***p < 0.001 in comparison with DMSO

To evaluate cytotoxicity, we determined the viability of MDCK cells after incubation for 24, 72, or 96 h in infection medium containing BSA using the MTT assay (Fig. 2). The viability of MDCK cells treated with H_2_O, EtOAc, Hex, or CHCl_3_ extract from the stems of *J. multifida* was unaffected after 24 h, compared with cells exposed to DMSO only (Fig. 2a). After 72 or 96 h of incubation, the viability of MDCK cells treated with 100 μg/mL H_2_O or 12.5-100 μg/mL CHCl_3_ extracts significantly reduced (Fig. 2b), whereas the viability of cells exposed to ≤ 50 μg/mL H_2_O, ≤ 100 μg/mL EtOAc and Hex, or ≤ 12.5 μg/mL CHCl_3_ extracts was unaffected compared with cells exposed to DMSO only (Fig. 2c). Therefore, these data suggested that exposure to ≤ 100 μg/mL H_2_O, EtOAc, Hex, or CHCl_3_ extract for 24 h, or ≤ 50 μg/mL H_2_O, ≤ 100 μg/mL EtOAc and Hex, or ≤ 12.5 μg/mL CHCl_3_ extracts for 72 or 96 h was not cytotoxic in MDCK cells.

Together, these results proved that the H_2_O extract from the stems of *J. multifida* promoted the survival of MDCK cells infected with the influenza A H1N1 virus. The EtOAc and CHCl_3_ extracts demonstrated similar, but weaker, effects.

### The extracts inhibited influenza A viral infection and growth

**Fig. 3:**
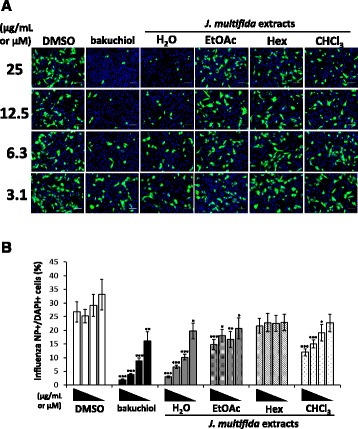
Extracts from the stems of *J. multifida* inhibited influenza A H1N1 viral infection. H_2_O, EtOAc, Hex, or CHCl_3_ extracts from the stems of *J. multifida* (3.1-25 μg/mL) (*n* = 9 each) or (+)-(*S*)-bakuchiol (bakuchiol; 3.1-25 μM) (*n* = 9) were mixed with influenza A virus (A/PR/8/34) at a MOI of 0.1 and added to MDCK cells. DMSO (0.031-0.25%) (*n* = 9) was used as the negative control. After 24 h, the cells were fixed and permeabilized. To visualize influenza A virus-infected cells, we performed immunofluorescent staining of influenza A viral NP (*green*) and cell nuclei (*blue*), using the nuclear-staining compound, DAPI. Cells were subsequently photographed under a fluorescence microscope (**a**), and the percentage of influenza A viral NP-positive cells per DAPI-positive cells was calculated based on influenza A viral NP-positive and DAPI-positive cell numbers (**b**). The white scale bar in each image represents 100 μm. Data are presented as means ± SEM of three independent experiments. *p < 0.05; **p < 0.01; ***p < 0.001 in comparison with DMSO

To investigate whether the extracts inhibited viral infection, we examined viral NP-immunofluorescence staining in MDCK cells treated with a mixture of virus and H_2_O, EtOAc, Hex, or CHCl_3_ extract for 24 h. The wells were observed under a microscope and photographed (Fig. 3a). NP-immunostained cells were counted, and the percentage of influenza A NP-positive cells per DAPI-positive cells was calculated (Fig. 3b). The percentage of influenza A NP-positive cells was significantly decreased in a concentration-dependent manner in samples treated with H_2_O, EtOAc, or CHCl_3_ extract or (+)-(*S*)-bakuchiol (positive control), compared with DMSO-treated cells (Fig. 3a and b). The H_2_O extract produced greater inhibition of influenza A viral infection than the other extracts. These data proved the inhibitory effect of the H_2_O, EtOAc, and CHCl_3_ extracts from the stems of *J. multifida* on influenza A virus H1N1 infection.

**Fig. 4:**
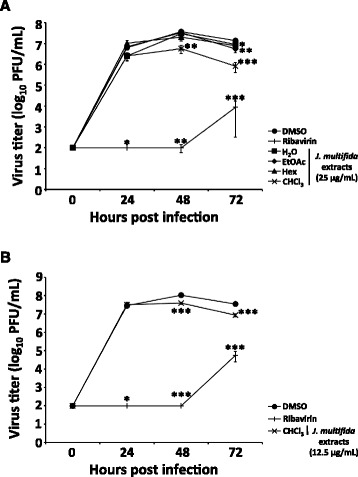
Extracts from the stems of *J. multifida* inhibited influenza A H1N1 viral growth. MDCK cells were infected with A/PR/8/34 (MOI; 0.001) for 1 h, and then the infected cells were washed. H_2_O, EtOAc, Hex, or CHCl_3_ extracts from the stems of *J. multifida* (25 μg/mL in 0.5% DMSO) (*n* = 7 each) (**a**) were added to the cells in the infection medium supplemented with 3 μg/mL TPCK-treated trypsin. DMSO (0.5%) (*n* = 7) or ribavirin (50 μM in 0.5% DMSO) (*n* = 7) were the negative and positive controls, respectively, for the inhibition of influenza A viral growth. In addition, the same experiment was performed with DMSO (0.5%), ribavirin (50 μM in 0.5% DMSO), and 12.5 μg/mL CHCl_3_extract from the stems of *J. multifida* (in 0.5% DMSO) (*n* = 12 each) (**b**). The conditioned culture medium was collected at the indicated time points, added to MDCK cells, and the treated cells were immunostained with an antibody to influenza A viral NP. The viral titers (PFU/mL) were calculated from the number of stained cells. The data are the mean ± SEM representative of three independent experiments. *p < 0.05; **p < 0.01; ***p < 0.001 in comparison with DMSO

Next, we investigated the inhibition of viral growth by the H_2_O, EtOAc, Hex, or CHCl_3_ extract for 24–72 h in virus-infected MDCK cells. The viral titers in conditioned media from samples treated with CHCl_3_ extract between 48 and 72 h, EtOAc or Hex extracts at 72 h, and ribavirin between 24–72 h were significantly decreased compared with those in media conditioned by DMSO-treated cells (Fig. 4a). However, owing to the cytotoxicity observed following a 72-h exposure of MDCK cells to 25 μg/mL CHCl_3_ extract in the viral growth experiment (Fig. 2b), we repeated the experiment with 12.5 μg/mL CHCl_3_ extract. The viral titers in the conditioned media from cells treated with 12.5 μg/mL CHCl_3_ extract at 48 and 72 h significantly decreased compared with those in the media conditioned by DMSO-treated cells (Fig. 4b). These data proved that the EtOAc, Hex, and CHCl_3_ extracts inhibited the growth of influenza A H1N1 virus, and that the CHCl_3_ extract possessed the strongest activity.

Together, these results demonstrated the inhibition of influenza A viral infection and growth by extracts from the stems of *J. multifida*. The variation in the inhibitory effects of the extracts may be due to the polarities of the phytochemical constituents present in the stems.

## Discussion

In the present study, the H_2_O extract from the stems of *J. multifida* strongly increased survival of influenza A virus-infected MDCK cells (Fig. 1) and inhibited influenza A viral infection (Fig. 3), whereas the CHCl_3_ extract demonstrated the strongest inhibition of influenza A viral growth (Fig. 4) compared with the other crude extracts. These results indicated that the different polarities of the H_2_O and CHCl_3_ crude extracts produced inhibition of viral infection or growth by different mechanisms. Infection with the influenza virus begins with binding to the surface of a host cell by viral hemagglutinin (HA), viral surface protein [19]. The influenza virus invades host cells by endocytosis, and the viral genome is then released into the host’s cytoplasm through fusion of the viral membrane with the host endosomal membrane via HA cleavage. The influenza viral genome replicates in the host nucleus using viral RNA polymerase. The virions bud and are released from the membrane of the host cell using viral NA. In the cell viability and viral infection assays, the H_2_O extract from the stems of *J. multifida* inhibited influenza A viral binding to host cells when the influenza virus and extracts were co-incubated in advance. In contrast, in the viral growth assay, the CHCl_3_ extract inhibited influenza A viral replication in host cells. We therefore propose that the H_2_O extract may include compounds that inhibit influenza A viral binding to host cells surface, endocytosis, membrane fusion, or uncoating by inhibiting viral HA, while the CHCl_3_ extract may include compounds that inhibit influenza viral replication in host cells by inhibiting viral RNA polymerase or NA activities.

Previously, chemical studies of the stems of *J. multifida* led to the isolation of lathyrane-type diterpenoids [20–24], jatrophane-type diterpenoids [11, 22], and coumarino-type lignoids [22]. The lathyrane-type diterpenoids, multifidone, multifidanol, and multifidenol, showed cytotoxicity and antibacterial activity [24]. The jatrophane-type diterpenoid, jatrophone, was reported to possess a wide range of biological effects such as cytotoxicity and antitumor activity [25, 26]. However, the anti-influenza activity of the phytochemical constituents of *J. multifida* has not been yet investigated. The lathyrane-type diterpenoids from *Euphorbia micractina* showed the anti-HIV activity [27]. Dang et al. recently reported that the abietane-type tricyclic phenolic diterpenoids, (+)-podcarpic acid and (+)-totarol, inhibited influenza A H1N1 viral infection (A/PR/8/34) [28]. Therefore, the diterpenoids contained in *J. multifida* may confer the anti-viral effects such as anti-influenza and anti-HIV activities. It is expected that anti-influenza compounds will be isolated from the active crude extracts in our ongoing work.

## Conclusions

The findings of the present study demonstrated that the most polar extract, the H_2_O extract, from the stems of *J. multifida* increased survival of MDCK cells infected with the influenza A H1N1 virus and showed the strongest inhibition of influenza A H1N1 viral infection in MDCK cells. Of the EtOAc, Hex, and CHCl_3_ extracts, the CHCl_3_ extract showed the strongest inhibition of influenza A H1N1 viral growth in MDCK cells. These results indicated that the stems of *J. multifida* could be used as a herbal medicine for the treatment of influenza and may be a source of candidate compounds for novel anti-influenza drug development.

## Abbreviations

ANOVA: One-way analysis of variance
CHCl_3_: Chloroform
DAPI: Diamidino-2-phenylindole
DMSO: Dimethyl sulfoxide
EtOAc: Ethyl acetate
H_2_O: Water
Hex: *n*-hexane
MDCK: Madin-Darby canine kidney
MTT: Thiazolyl blue tetrazolium bromide
NA: Neuraminidase
NP: Nucleoprotein
P/S: Penicillin and streptomycin
PBS: Phosphate-buffered saline
PR: Puerto Rico
TPCK: l-tosylamido-2-phenyl ethyl chloromethyl ketone
